# Supplementary material for: Effect of Vesicle Size on the Cytolysis of Cell-Penetrating Peptides (CPPs)
Source: Int J Mol Sci. 2020 Oct 7;21(19):7405. doi: 10.3390/ijms21197405 (PMC7582850; doi:10.3390/ijms21197405)

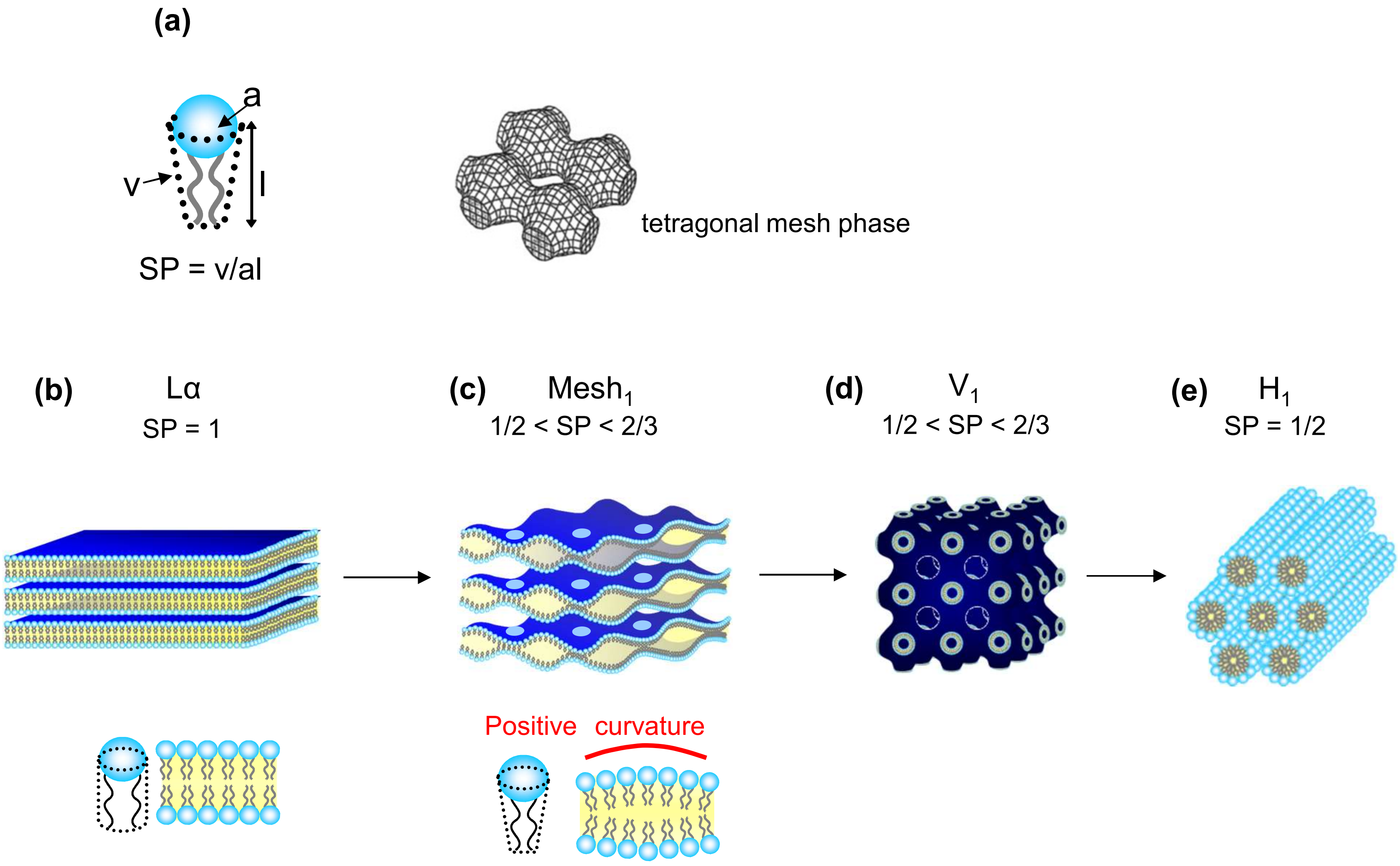

(a)
a
v
l
SP = v/al
tetragonal mesh phase
(b)
Lα
SP = 1
(c)
Mesh$_1$
1/2 < SP < 2/3
(d)
V$_1$
1/2 < SP < 2/3
(e)
H$_1$
SP = 1/2
Positive curvature

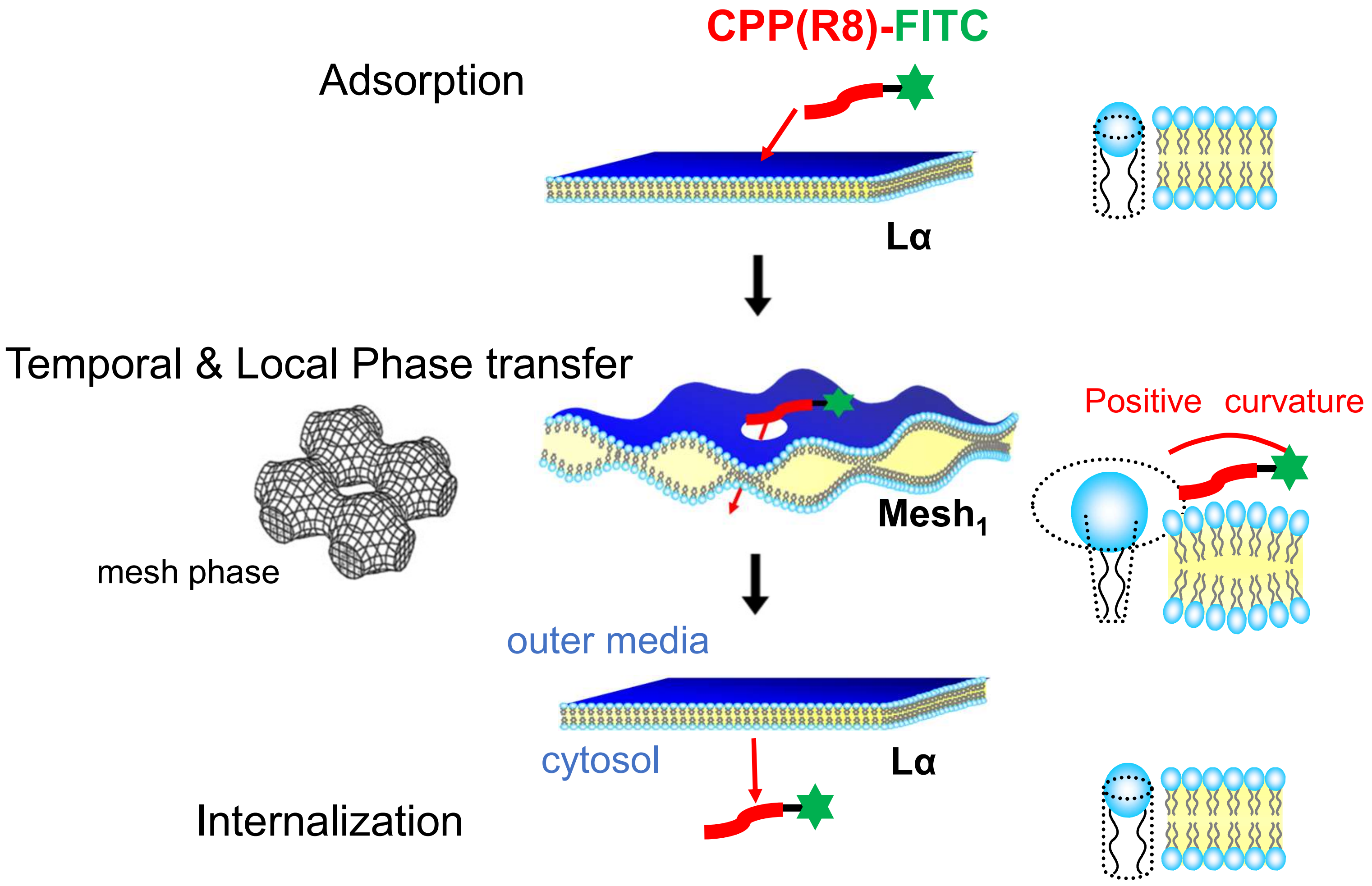

Cytolysis mechanism (Direct internalization)

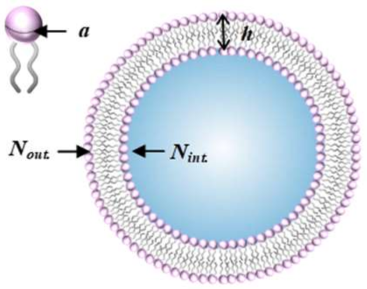

The number of phospholipid molecules of the outer surface of the vesicles (N_{out}) is represented by the formula (3).

$$N_{out} = \frac{4\pi\left(\frac{d}{2}\right)^2}{a} \quad (3)$$

The number of phospholipid molecules of the internal surface of the vesicles (N_{int}) is represented by the formula (4).

$$N_{int} = \frac{4\pi\left(\frac{d}{2} - h\right)^2}{a} \quad (4)$$

Since the molecular occupation area a of E-PC is 0.71nm^2 and the thickness the bi-layer formed by the phospholipid is about 5nm, the number of phospholipid molecules consisting GUV of E-PC is represented by the following formula (5).

$$N_{tot} = \frac{\left[4\pi\left(\frac{d}{2}\right)^2 + 4\pi\left(\frac{d}{2} - 5\right)^2\right]}{a} \quad (5)$$

Since the diameter of GUV is about 14μm, N_{tot}=1.7 X 10^9 by formulae (5), N_{out}=8.8 X 10^8 from formulae (4) and N_{int}=8.8 X 10^8 are obtained.

The number of the vesicles N_{vesic} in 1mL vesicle suspension is represented by the following formula (6).

$$N_{vesic} = \frac{M_{lipid} \times N_A}{N_{tot} \times 1000} \quad (6)$$

Wherein M_{lipid} and N_A represent molar concentration (mol/L) of the phospholipid and the Avogadro number (6.0 X 10^{23}(mol^{-1})), respectively.

Since the molar concentration of the phospholipid is 1.7mM, N_{vesi} is obtained by the above formula (6) as follows.

N_{vesi} =5.82 X 10^8 (unit/mL).

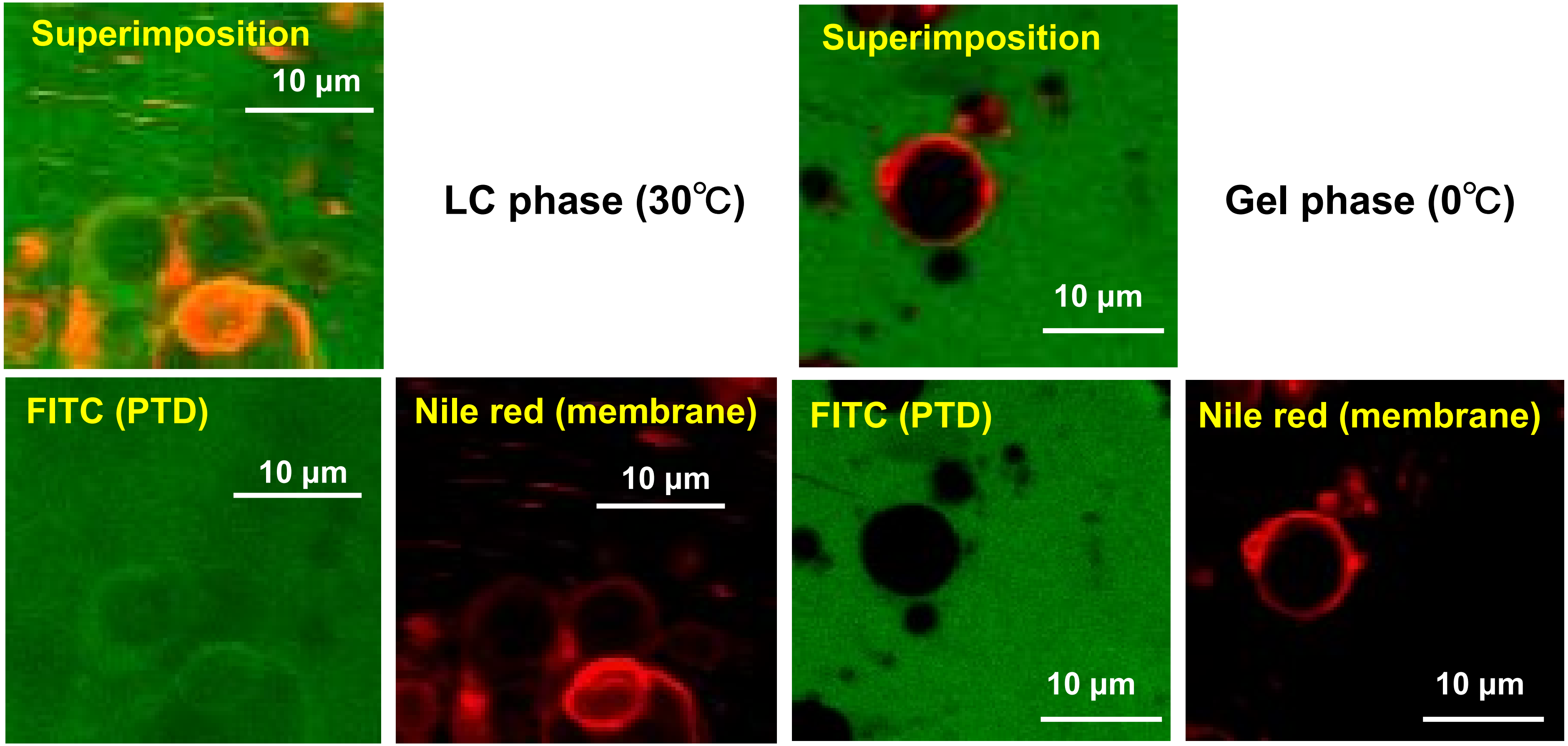
Superimposition
10 μm
LC phase (30℃)
Superimposition
10 μm
Gel phase (0℃)
FITC (PTD)
10 μm
Nile red (membrane)
10 μm
FITC (PTD)
10 μm
Nile red (membrane)
10 μm

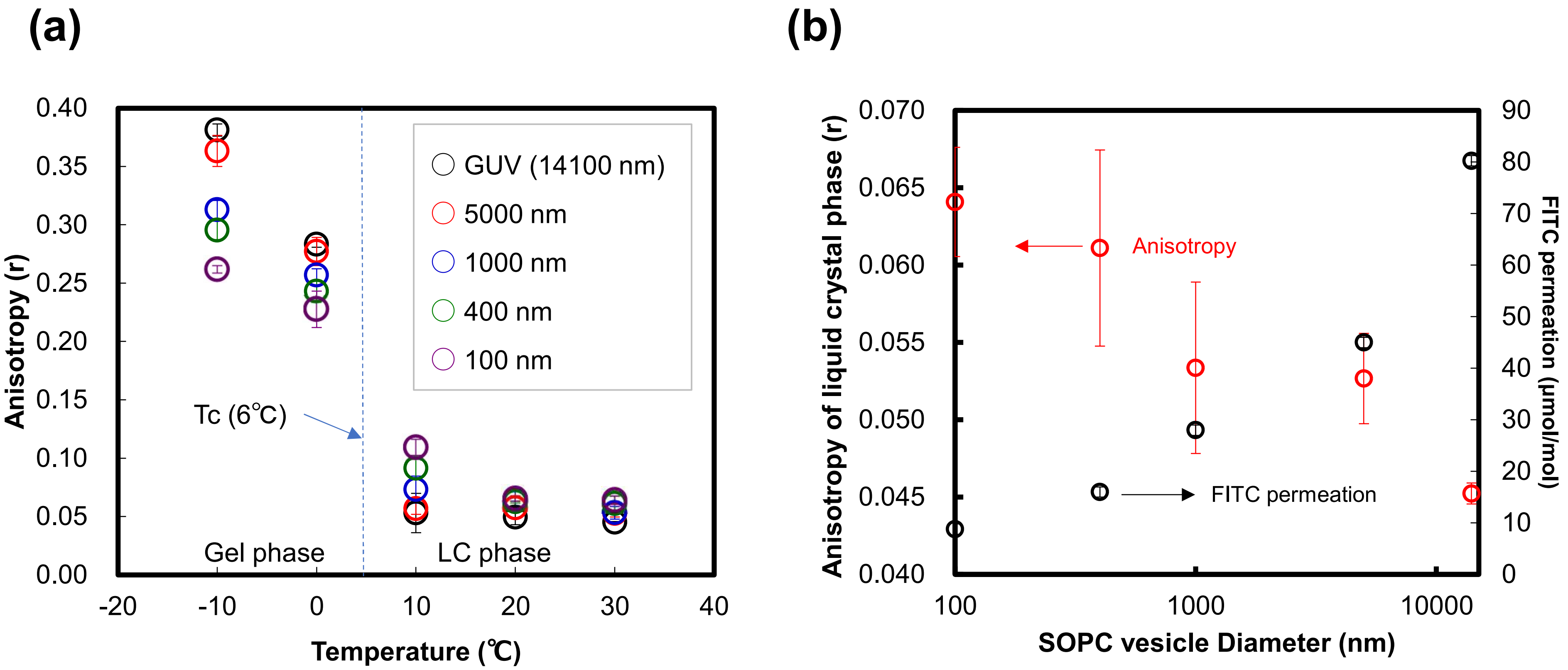
(a)
Anisotropy (r)
0.40
0.35
0.30
0.25
0.20
0.15
0.10
0.05
0.00
-20
-10
0
10
20
30
40
Temperature (℃)
GUV (14100 nm)
5000 nm
1000 nm
400 nm
100 nm
Tc (6℃)
Gel phase
LC phase
(b)
Anisotropy of liquid crystal phase (r)
0.070
0.065
0.060
0.055
0.050
0.045
0.040
FITC permeation (μmol/mol)
90
80
70
60
50
40
30
20
10
0
100
1000
10000
SOPC vesicle Diameter (nm)
Anisotropy
FITC permeation

Conceptual model for CPC cytolysis

"The Man Who Could Walk through Walls"   (Le Passe-Muraille)
By Marcel Aymé, 1943 [21]

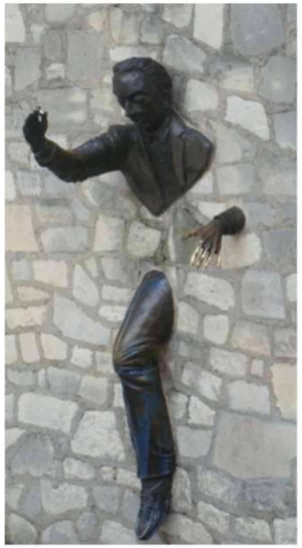

Statue at Montmartre, Paris
Courtesy of Dr. Nomura, Hokkaido Univ.

One day Mr. Dutillueul (Mr. D) discovered himself capable to walk through walls . When he approaches, he felt wall surrounding him melts into fluid and he can walk in and through it without any resistance.

Cell membrane as a wall for CPP (Mr. D) must be permeable fluid to be passed through.
If the wall is resistant to change its curvature, Mr. D must have difficulty to walk into the wall.

Cytolysis mechanism (Direct internalization) [12]

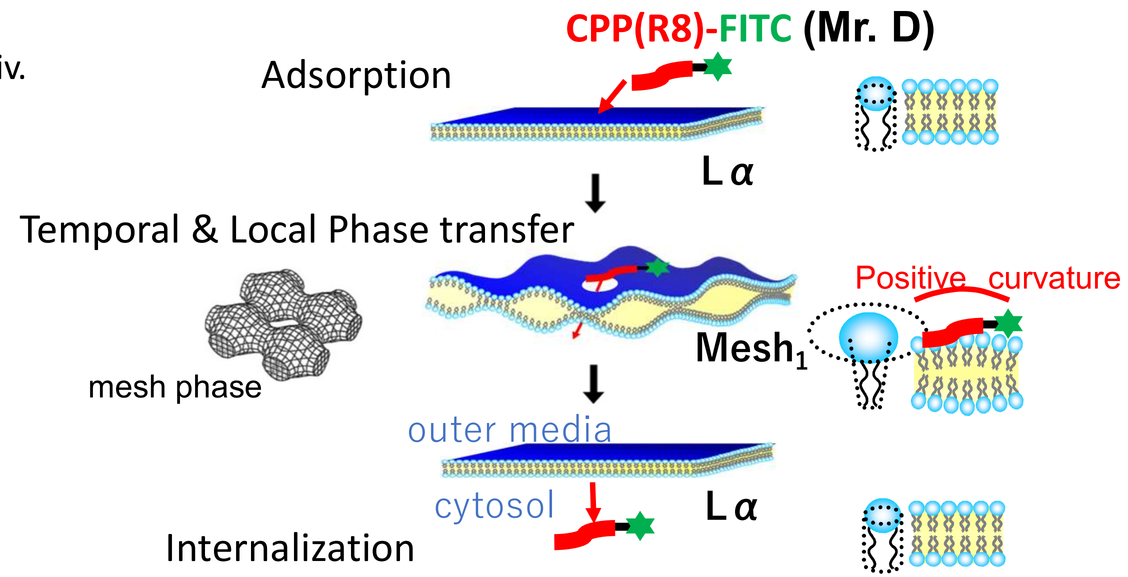

Supplement: Supplementary file 1 [file ijms-21-07405-s001.pdf]
